# Retroperitoneal inflammatory myofibroblastic tumor

**DOI:** 10.1186/1477-7819-3-66

**Published:** 2005-10-08

**Authors:** Suresh VS Attili, C Rama Chandra, Dadhich K Hemant, Poonamalle P Bapsy, Clementeena RamaRao, G Anupama

**Affiliations:** 1 Department of Medical Oncology, Kidwai Memorial Institute of Oncology, Bangalore, India; 2 Department of SurgicalOncology, Kidwai Memorial Institute of Oncology, Bangalore, India; 3 Department of Pathology, Kidwai Memorial Institute of Oncology, Bangalore, India

## Abstract

**Background:**

Inflammatory myofibroblastic tumor (IMT) is a neoplasm of unknown etiology occurring at various sites. By definition, it is composed of spindle cells (myofibroblasts) with variable inflammatory component, hence the name is IMT.

**Case presentation:**

The present case is of a 46 years old woman presented with a history of flank pain, abdominal mass and intermittent hematuria for last 6 months. The initial diagnosis was kept as renal cell carcinoma. Finally, it turned out to be a case of retroperitoneal IMT. The patient was managed by complete surgical resection of the tumor.

**Conclusion:**

IMT is a rare neoplasm of uncertain biological potential. Complete surgical resection remains the mainstay of the treatment.

## Introduction

Inflammatory myofibroblastic tumor (IMT) is a relatively rare neoplasm. The outlook of this disease has changed with time from a benign reactive process to a malignant neoplasm, based on the multiple case reports demonstrating recurrent and constant clonal genetic alterations [1-5]. There are three main histological patterns: nodular fasciitis-like, fibrous histiocytoma-like, and desmoid or scar tissue-type. Though morphologically similar, they encompass a spectrum of entities with varied etiology, ranging from reactive/regenerative proliferations to low-grade neoplasms with a risk of local recurrence, and metastatic potential [3]. The commonest site of IMT is lung with a few case reports from extra pulmonary sites [6]. In the genitourinary tract, it most commonly occurs in the bladder. However it rarely originates in the kidney, renal pelvis, and ureter [4]. In the English literature only six cases of retroperitoneal IMT were reported [6-8]

## Case presentation

**Figure 1:**
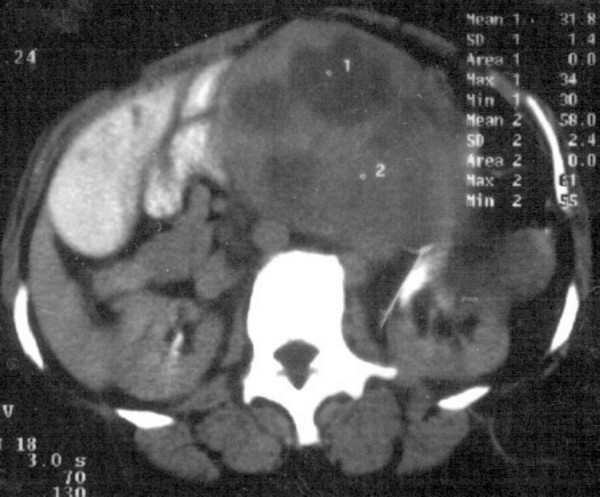
CT abdomen of the patient showing large irregular heterogeneous lesion measuring approximately 16 × 13.6 × 12.1 cm with predominant cystic portions without calcifications.

**Figure 2:**
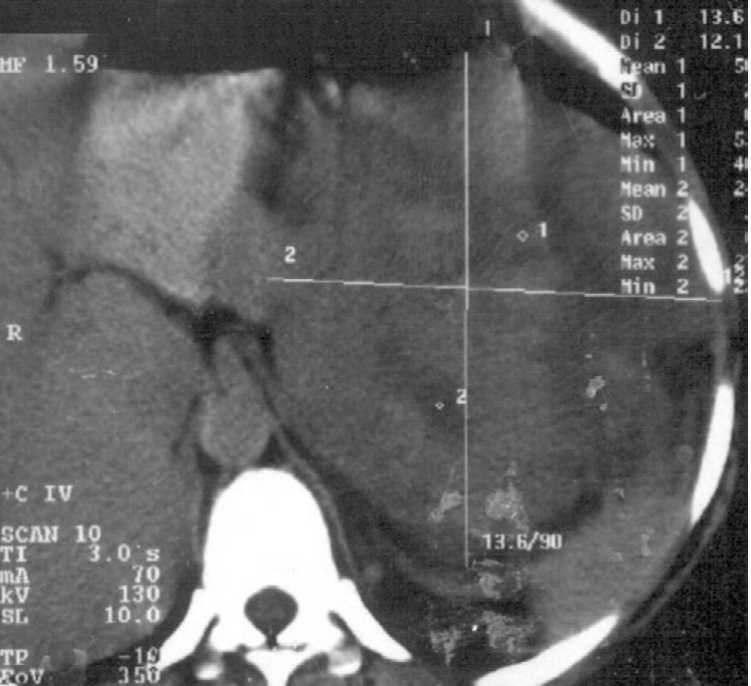
contrast enhanced CT abdomen of the patient showing the same lesion as in fig. 1 which is non enhancing.

**Figure 3:**
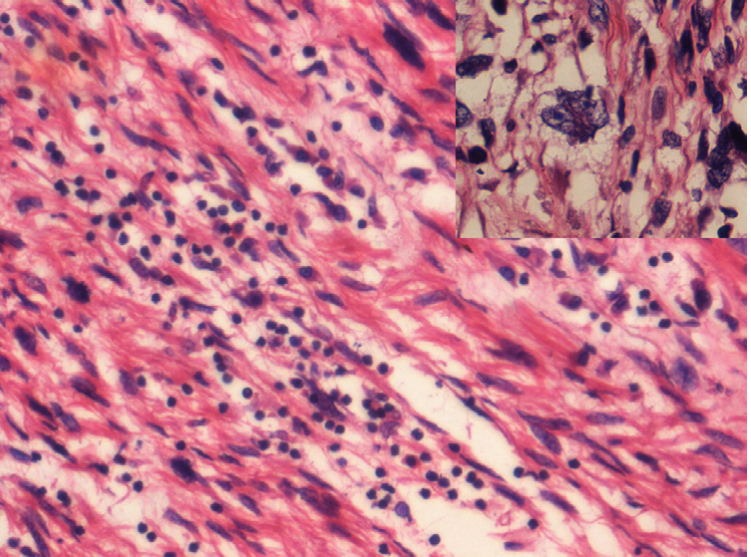
Spindle cells with interspersed lymphocytes and plasma cells. Inset shows pleomorphic, multilobed tumor cells (Hematoxylin and Eosin × 400).

A 46-years-old woman presented with history of flank pain, abdominal lump and intermittent hematuria of 6 months duration. She was diagnosed as renal cell carcinoma (RCC) elsewhere and referred. The investigations at our hospital revealed normal hematological and biochemical parameters. The urine microscopy showed deformed RBC. The computerized tomography (CT) scan of the abdomen showed a large irregular well defined heterogeneous lesion occupying the left hypochondrium, lumbar and supra umbilical regions, measuring approximately 16 × 13.6 × 12.1 cm, and showed presence of predominant cystic portions with no obvious calcifications (figure 1 &2). The mass was contiguously involving the upper half of the kidney. Fine needle aspiration cytology (FNAC) of the mass was suggestive of sarcomatoid renal cell carcinoma. The metastatic work-up including bone scan, and CT scan of the thorax were essentially normal. With a provisional diagnosis of renal cell carcinoma surgery was planned. Intra operatively a huge mass was observed in the retroperitoneal region arising from the left kidney and attempts to separate the mass from the kidney were unsuccessful. Hence, left nephrectomy with adrenalectomy was performed with complete excision of the tumor mass. Histopathology showed large encapsulated mass 16 × 17 × 12 cm attached to kidney on its posterior aspect. The tumor showed a predominantly spindle cell pattern consisting of cellular fascicles showing mild atypia, with a diffuse sprinkling of lymphocytes and plasma cells and a few lymphoid aggregates. Only an occasional mitosis was identified in these areas. Hypocellular areas with sparse cells and a collagenous stroma were also present. One area of the tumor showed features of malignant transformation, with sheets of bi and multinucleated and multilobed cells in a background of atypical spindle cells with interspersed inflammatory cells (figure 3). Mitoses in this area were 3–4/high power field and focal necrosis was present. Immunohistochemisty for desmin, smooth muscle actin (SMA), epithelial membrane antigen (EMA), cytokeratin (CK), CD68, HMB45 and ALK were performed. Desmin showed diffuse positivity, while SMA, EMA and CD68 showed focal positivity. CK, HMB45 and ALK were negative. A diagnosis of inflammatory myofibroblastic tumor with malignant transformation was made.

## Discussion

IMT is a relatively rare neoplasm. It was previously referred as plasma cell granuloma, or inflammatory pseudo tumor (IPT). It has long been debated regarding the origin of IMT whether it was truly neoplastic or a post inflammatory process. The proposed etiologies included Epstein Barr virus (EBV), Human herpes virus (HHV8), and over expression of interleukin IL-6. Though other diseases like Kaposi's sarcoma and Castleman's diseases also have similar etiologies, molecular transcription form of open reading frame (ORF) -16, K13, 72 expressed in IMT are not expressed in the aforesaid diseases [9]. Moreover the recent research suggest that IMT is probably a neoplasm rather than a post-inflammatory process because of cytogenetic clonality, recurrent involvement of chromosomal region 2p23, occasional aggressive local behavior and metastasis of the tumor [1,2,4,5].

It is more common in children without sex predilection, though extra pulmonary forms are more common in adult females. The patients often present with fever of unknown origin and other vague nonspecific symptoms. Usually it has a benign course and in most of the cases it is a slow growing, locally confined tumor with less metastatic potential. However, there are some predictors for aggressive behavior and metastatic potential of IMT which include presence of ganglion like cells, cellular atypia, aneuploidy, and p53 over expression [11,12].

The tumor commonly occurs in lung. Extra pulmonary IMT are rare. In the largest series of 84 cases of extra pulmonary IMT, only four retroperitoneal IMT were reported [6] The medline search revealed only six cases of exclusive retroperitoneal IMT (excluding renal and pancreatic IMT). All the cases had flank pain as in our case. However hematuria which was observed in our case was found only in one case [8]. Other common features like fever, weight loss were not observed in our case. We initially approached the case as renal cell carcinoma due to the presence of classical triad of pain, lump and hematuria along with typical CT scan findings, and to our surprise the final diagnosis came as IMT. It would be possible on frozen section to differentiate this tumor from renal cell carcinoma. However the area showing malignant transformation could be confused with a sarcomatoid renal cell carcinoma. It is also difficult on frozen section to differentiate this tumor from an inflammatory process and this difficulty would also apply to assessment of margins of resection. So we feel that the total excision was more appropriate as the tumor is known for more local recurrences than distant metastasis. Preoperative or intra operative FNAC would be adequate to differentiate this lesion from renal cell carcinoma. However if the pleomorphic area is sampled it could lead to confusion with a sarcomatoid RCC. Therefore, avoiding of this situation is difficult and whenever the suggestion is IMT, a complete excision is advisable rather than going for organ preserving surgery.

Though IMT will not make its place in the common differential diagnosis of retroperitoneal masses, it should be kept in mind as one of the possibilities. Typically the IMT is characterized by the expression of vimentin, smooth muscle actin, and cytokeratins, corresponded to that of myofibroblasts along with other inflammatory markers [10]. In the present case, the IHC is positive for EMA and desmin, which are smooth muscle markers and CD-68 is an inflammatory marker, suggesting the tumor to be IMT. Little data is available in the management of IMT owing to its rarity. Surgery remains the main stay of treatment. Though radiotherapy [12], immunosuppression and chemotherapy (cisplatin, doxorubicin & methotrexate) [13] have been tried as an adjunct to surgery, however, no definitive benefit was demonstrated by any of these modalities [14]. In the present case as the tumor was completely removed and the metastasis is rare in IMT, the patient was offered no adjuvant therapy. The patient remained asymptomatic even after 1 year of follow-up without any evidence of disease. We expect long term survival in this case, as all the reported cases of retroperitoneal IMT had long disease-free survival [8].

## Competing interests

The author(s) declare that they have no competing interests.

## Authors' contributions

**SVSA **have been involved in drafting the manuscript & revising it critically for important intellectual content

**RCC **acquisition of data, or analysis and interpretation of data

**HKD **revising it critically for important intellectual content

**PPB **revising it critically for important intellectual content and given final approval of the version to be published

**CRR **interpretation of data

**GA **have been involved in drafting the manuscript & revising it critically for important intellectual content

## References

[B1] Pungpapong S, Geiger XJ, Raimondo M (2004). Inflammatory myofibroblastic tumor presenting as a pancreatic mass: a case report and review of the literature. JOP.

[B2] Coffin CM, Dehner LP, Meis-Kindblom JM (1998). Inflammatory myofibroblastic tumor, inflammatory fibrosarcoma, and related lesions: an historical review with differential diagnostic considerations. Semin Diagn Pathol.

[B3] Freeman A, Geddes N, Munson P, Joseph J, Ramani P, Sandison A, Fisher C, Parkinson MC (2004). Anaplastic lymphoma kinase (ALK 1) staining and molecular analysis in inflammatory myofibroblastic tumors of the bladder: a preliminary clinicopathological study of nine cases and review of the literature. Mod Pathol.

[B4] Kapusta LR, Weiss MA, Ramsay J, Lopez-Beltran A, Srigley JR (2003). Inflammatory myofibroblastic tumors of the kidney: a clinicopathologic and immunohistochemical study of 12 cases. Am J Surg Pathol.

[B5] Biselli R, Boldrini R, Ferlini C, Boglino C, Inserra A, Bosman C (1999). Myofibroblastic tumors: neoplasias with divergent behavior. Ultrastructural and flow cytometric analysis. Pathol Res Pract.

[B6] Coffin CM, Watterson J, Priest JR, Dehner LP (1995). Extrapulmonary inflammatory myofibroblastic tumor (inflammatory pseudotumor). A clinic pathologic and immuno-histochemical study of 84 cases. Am J Surg Pathol.

[B7] Esmer-Sanchez D, Rangel D (2002). Inflammatory pseudotumor of the retroperitoneum. Rev Gastroenterol Mex.

[B8] Tambo M, Kondo H, Kitauchi T, Hirayama A, Cho M, Fujimoto K, Yoshida K, Ozono S, Hirao Y, Yamada E, Ichijima K (2003). A case of inflammatory myofibroblastic tumor of the retroperitoneum. Hinyokika Kiyo.

[B9] Gomez-Roman JJ, Sanchez-Velasco P, Ocejo-Vinyals G, Hernandez-Nieto E, Leyva-Cobian F, Val-Bernal JF (2001). Human herpesvirus-8 genes are expressed in pulmonary inflammatory myofibroblastic tumor (inflammatory pseudotumor). Am J Surg Pathol.

[B10] Sastre-Garau X, Couturier J, Derre J, Aurias A, Klijanienko J, Lagace R (2002). Inflammatory myofibroblastic tumour (inflammatory pseudotumour) of the breast. Clinicopathological and genetic analysis of a case with evidence for clonality. J Pathol.

[B11] Hussong JW, Brown M, Perkins SL, Dehner LP, Coffin CM (1999). Comparison of DNA ploidy, histological and immunohistochemical findings with clinical outcome in inflammatory myofibroblastic tumors. Mod Pathol.

[B12] Imperato JP, Folkman J, Sagerman RH, Cassady JR (1986). Treatment of plasma cell granuloma with radiation therapy: a report of two cases and a review of the literature. Cancer.

[B13] Dishop MK, Warner BW, Dehner LP, Kriss VM, Greenwood MF, Geil JD, Moscow JA (2003). Successful treatment of inflammatory myofibroblastic tumor with malignant transformation by surgical resection and chemotherapy. J Pediatr Hematol.

[B14] Hagenstad CT, Kilpatrick SE, Pettenati MJ, Savage PD (2003). Inflammatory myofibroblastic tumor with bone marrow involvement. A case report and review of the literature. Arch Pathol Lab Med.

